# Interaction of triosephosphate isomerase from the cell surface of *Staphylococcus aureus* and *α*-(1→3)-mannooligosaccharides derived from glucuronoxylomannan of *Cryptococcus neoformans*

**DOI:** 10.1099/mic.0.028068-0

**Published:** 2009-08

**Authors:** Hiromi Furuya, Reiko Ikeda

**Affiliations:** Department of Microbiology, Meiji Pharmaceutical University, 2-522-1 Noshio, Kiyose, Tokyo 204-8588, Japan

## Abstract

The glycolytic enzyme triosephosphate isomerase (TPI; EC 5.3.1.1) of *Staphylococcus aureus* is a candidate adhesion molecule for the interaction between the bacterium and the fungal pathogen *Cryptococcus neoformans*. TPI may recognize the mannan backbone of glucuronoxylomannan (GXM) of *C. neoformans*. We purified TPI from extracts of *S. aureus* surface proteins to investigate its binding by surface plasmon resonance analysis. The immobilized TPI reacted with GXM in a dose-dependent manner. Furthermore, the interactions between staphylococcal TPI and *α*-(1→3)-mannooligosaccharides derived from GXM were examined. The oligosaccharides exhibited binding with TPI; however, monomeric mannose did not. Differences in the slopes of the sensorgrams were observed between oligosaccharides with an even number of residues versus those with an odd number. A heterogeneous ligand-parallel reaction model revealed the existence of at least two binding sites on TPI. The enzymic activities of TPI were inhibited in a dose-dependent manner by *α*-(1→3)-mannooligosaccharides larger than triose. The binding of TPI and *α*-(1→3)-mannotriose near the substrate-binding site was predicted *in silico* (AutoDock 3.05). An oligosaccharide of size equal to or greater than triose could bind to the site, affecting enzymic activities. Moreover, affinities were indicated, especially for biose and tetraose, to another binding pocket, which would not affect enzymic activity. These data suggest a novel role for TPI, in addition to glycolysis, on the surface of *S. aureus*.

## INTRODUCTION

The search for novel targets for the regulation of microbes has focused on interactions between micro-organisms. Diffusible signalling molecules that play essential roles in bacterial cell–cell communication have been identified (Kaper & Sperandio, 2005; Ryan & Dow, 2008; von Bodman *et al.*, 2008). Concentrating on the fungal−bacterial interaction, we previously reported contact-mediated killing of *Cryptococcus neoformans* by *Staphylococcus aureus* (Saito & Ikeda, 2005).

*Cryptococcus neoformans*, an encapsulated yeast pathogen that causes severe meningitis, is found predominantly in avian excreta, especially that of the pigeon. This fungus is characterized by a capsule composed mainly of the acidic heteropolysaccharide glucuronoxylomannan (GXM), which has a backbone consisting of *α*-1,3-mannan with a single branch of *β*-1,2-xylose or glucuronic acid per mannose residue. *C. neoformans* enters the human body via the respiratory pathway (Casadevall & Perfect, 1998). *S. aureus* is found in the nasal cavities of a large percentage of normal healthy subjects (Kluytmans *et al.*, 1997). In *S. aureus* infection, nasal colonization is a risk factor for invasive infection (von Eiff *et al.*, 2001; Foster, 2004; Park *et al.*, 2008), and the elimination of *S. aureus* nasal carriage reduces the development of the infection. However, the presence of *S. aureus* in the nasal mucosa may also provide a defence against the entry of *C. neoformans*.

Previously, we found that cell–cell contact is required for the apoptosis-like cell death of *C. neoformans* (Ikeda & Sawamura, 2008). The cell surface adhesion molecules involved in this interaction were identified as triosephosphate isomerase (TPI) and *α*-(1→3)-mannooligosaccharides larger than triose in *S. aureus* and *C. neoformans*, respectively (Ikeda *et al.*, 2007). In the present study, we purified staphylococcal TPI and examined its interactions with *α*-(1→3)-mannooligosaccharides derived from *C. neoformans* in order to characterize the binding affinities and predict binding models for TPI.

## METHODS

### Strain.

*Staphylococcus aureus* RN4220 was obtained as a gift from K. Sekimizu (University of Tokyo, Japan).

### Preparation of surface proteins from *S. aureus*.

Surface protein extracts from *S. aureus* were prepared as previously described (Ikeda *et al.*, 2007; Komatsuzawa *et al.*, 1997). Briefly, *S. aureus* cells that had been cultured in Trypticase soy broth (TSB; Becton Dickinson) at 37 °C for 6 h were suspended in 3 M LiCl. After mixing gently on ice for 15 min, the cell extract was dialysed against 10 mM phosphate buffer (pH 6.8) and the surface proteins were collected. TPI activity was not detected in the cellular debris remaining after surface protein isolation.

### TPI activity assay.

Enzymic activity was coupled to the oxidation of NADH by glycerol-3-phosphate dehydrogenase and measured as a change in absorbance at 340 nm, according to the method of Rozacky *et al.* (1971) with minor modifications. Briefly, *S. aureus* surface proteins were incubated with 0.45 mM dl-glyceraldehyde 3-phosphate as the substrate, 0.03 mg NADH and 3 μg glycerol-3-phosphate dehydrogenase in a final volume of 0.3 ml, and the *A*_340_ of the supernatant was monitored.

### Purification of TPI from *S. aureus*.

To purify TPI from the sample, ammonium sulfate at 50 % saturation was added followed by centrifugation to remove the precipitates, and the supernatant was dialysed against 50 mM phosphate buffer (pH 6.8). Additional ammonium sulfate (2.5 M) was added and the sample was applied to a column containing about 0.5 ml phenyl-Sepharose CL-4B. The protein was then eluted with a step gradient of 2.5−0 M ammonium sulfate in 50 mM phosphate buffer (pH 6.8). The fraction with TPI activity was dialysed against 10 mM phosphate buffer (pH 6.8) and the sample was applied to a column containing about 0.5 ml DEAE-Toyopearl 650M (Tosoh Bioscience). The protein was eluted with a step gradient of 0−1.0 M NaCl in 10 mM phosphate buffer (pH 6.8). The fraction with TPI activity was dialysed against PBS (pH 7.2) and the sample was applied to a Bio-Gel P-100 column (1.5×100 cm; Bio-Rad) for final purification.

### Molecular mass of TPI.

SDS-PAGE was performed at a constant current of 20 mA using a Multi Gel II mini 12.5 system (Cosmo Bio). After electrophoresis, the bands were silver stained. Briefly, the gel was fixed in 25 % (v/v) ethanol and 5 % (v/v) acetic acid for 3 h or overnight, washed twice for 10 min each with deionized water, incubated in sodium thiosulfate (40 mg in 200 ml) for 1 min, and washed twice for 1 min each with deionized water. The gel was then incubated with silver nitrite solution (200 mg in 200 ml) for 30 min, washed once for 30 s with deionized water, and developed for about 10 min with 2 % (w/v) sodium carbonate and 0.015 % (v/v) formaldehyde solution. The reaction was quenched with acetic acid, and the gel was washed three times for 5 min each with deionized water. The molecular mass of the purified protein was determined based on a set of protein markers (broad-range; ColourPlus Prestained Protein Marker, BioLabs).

### Preparation of oligosaccharides.

Oligosaccharides were prepared as previously described (Ikeda *et al.*, 2007). Briefly, the controlled Smith degradation product was hydrolysed with 0.4 M H_2_SO_4_ at 100 °C for 1 h. After neutralization with BaCO_3_, the product was applied to a Bio-Gel P-2 column (2.5×120 cm; Bio-Rad) and eluted with water (10 ml h^−1^, 2 ml fractions). Carbohydrate concentrations were determined by the phenol/sulfuric acid method. To identify mannooligosaccharides, the NMR spectra of the oligosaccharide fractions were recorded (Ichikawa *et al.*, 2001; Ikeda & Maeda, 2004).

### Surface plasmon resonance (SPR) analysis of the interaction between cryptococcal GXM and staphylococcal TPI.

**Fig. 1. f1:**
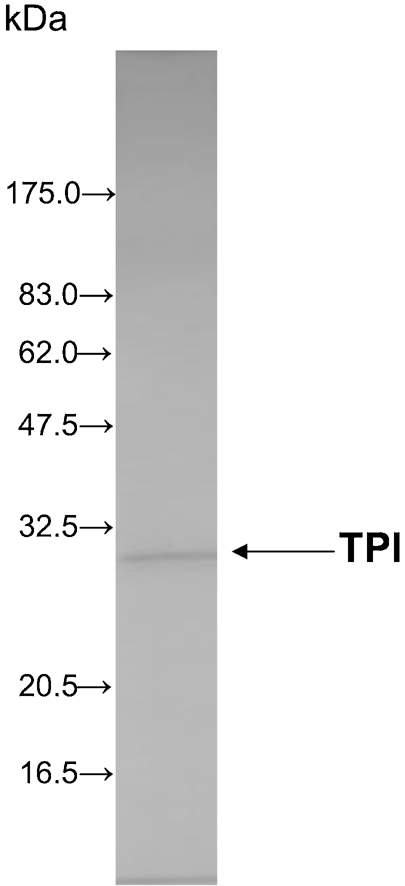
SDS-PAGE of purified TPI. After purification, TPI was confirmed by SDS-PAGE followed by silver staining. The molecular mass of this single band is consistent with that of TPI.

The interaction of *S. aureus* TPI and *C. neoformans* GXM was analysed using a Biacore 3000 biosensor system (GE Healthcare). The purified TPI, confirmed using SDS-PAGE followed by silver staining (Fig. 1), was diluted with 10 mM sodium acetate buffer (pH 3.48) and immobilized on a standard sensor chip CM 5 (GE Healthcare) using an amine coupling kit, according to the manufacturer's instructions. The analyte, GXM, was diluted with HBS-EP running buffer [10 mM HEPES (pH 7.4), 150 mM NaCl, 3 mM EDTA and 0.005 % (w/v) surfactant P20] and injected into the flow cell. The buffer flow rate was maintained at 10 μl min^−1^ for the immobilization and at 20 μl min^−1^ for the analysis.

### SPR analysis of the interaction between *α*-(1→3)-mannooligosaccharides and TPI.

The interactions between TPI and *α*-(1→3)-mannooligosaccharides derived from *C. neoformans* were determined as described in the preceding section, except that *α*-(1→3)-mannooligosaccharides were used as analytes. Each analyte was diluted with HBS-EP buffer and the buffer flow rate was maintained at 20 μl min^−1^.

### Docking simulation for mannotriose from *C. neoformans* and TPI from *S. aureus*.

The docking simulation of mannotriose and *S. aureus* TPI was performed using AutoDock 3.05 (Morris *et al.*, 1998). The PDB file of mannotriose was obtained from the Sweet website (http://www.dkfz-heidelberg.de/spec/) (Bohne *et al.*, 1999). The PDB file of TPI from *S. aureus* was obtained from the swiss-model website (Guex & Peitsch, 1997; Schwede *et al.*, 2003).

### Inhibition of TPI activity by mannooligosaccharides.

We investigated the possible inhibition of TPI activity by the mannooligosaccharides mannose, mannobiose, mannotriose, mannotetraose and mannopentaose. TPI activity in the presence of each mannooligosaccharide at 0, 10 and 100 μM was determined using the TPI assay described above.

## RESULTS

### Purification of TPI from *S. aureus*

As summarized in Table 1, TPI was purified from *S. aureus* surface proteins by salting out followed by hydrophobic, anion-exchange, and gel-filtration column chromatography. The purified TPI had a molecular mass of 28.5 kDa, as determined by SDS-PAGE (Fig. 1). The yield of 1.7 μg TPI reflected a 2400-fold purification.

### Interaction between GXM from *C. neoformans* and TPI from *S. aureus*

**Fig. 2. f2:**
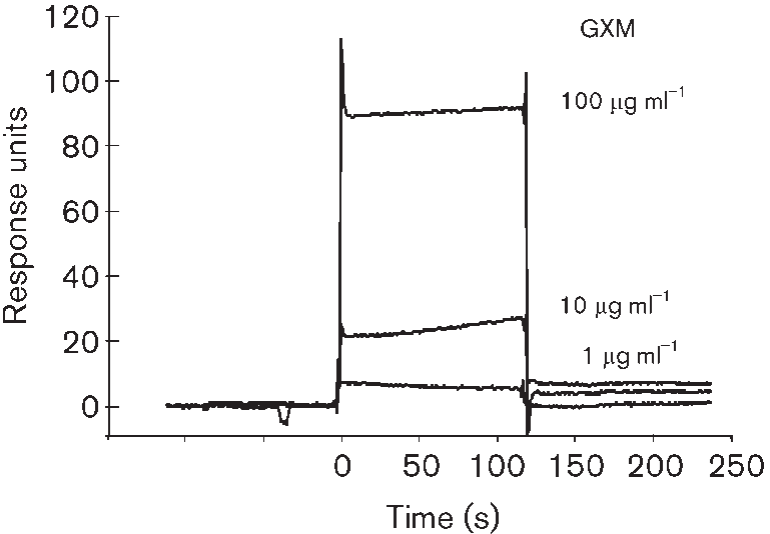
Sensorgrams from SPR analyses showing the interaction between TPI from *S. aureus* and GXM from *C. neoformans*. TPI from *S. aureus* was the ligand and GXM from *C. neoformans* was the analyte.

Fig. 2 shows the SPR sensorgrams for the interaction between GXM and immobilized TPI. The SPR response occurred in a dose-dependent manner.

### Interactions between *α*-(1→3)-mannooligosaccharides and TPI

**Fig. 3. f3:**
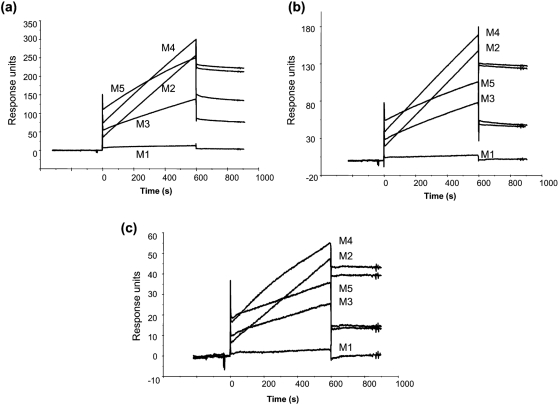
Sensorgrams from SPR analyses showing the interaction between TPI from *S. aureus* and *α*-(1→3)-mannooligosaccharides from *C. neoformans*. TPI from *S. aureus* was the ligand and *α*-(1→3)-mannooligosaccharides were the analytes. (a) 500 μM *α*-(1→3)-mannooligosaccharides, (b) 300 μM *α*-(1→3)-mannooligosaccharides, (c) 100 μM *α*-(1→3)-mannooligosaccharides. M1, mannose; M2, mannobiose; M3, mannotriose; M4, mannotetraose; M5, mannopentaose.

The SPR analyses revealed that the *α*-(1→3)-mannooligosaccharides derived from *C. neoformans* interacted with TPI from *S. aureus* in a dose-dependent manner (Fig. 3). The rate at which the response increased (expressed in response units) over the injection time (0–60 s) for each mannooligosaccharide decreased in the following order: mannopentaose, mannotetraose, mannotriose and mannobiose; mannose did not display interaction with TPI. However, from 60 to 600 s, the rate of increase in the response was greater for mannotetraose and mannobiose than for mannopentaose and mannotriose. The sensorgrams of the TPI interactions showed steeper slopes for oligosaccharides containing an even number of residues than for those with an odd number of residues, suggesting a difference in the mode of binding between the two types of oligosaccharides.

### Analysis and evaluation of the interactions between TPI and *α*-(1→3)-mannooligosaccharides

The association and dissociation kinetics of each mannooligosaccharide were evaluated using BIAevaluation version 4.1. Based on the sensorgrams, a 1 : 1 Langmuir binding model was not considered for the interaction between TPI and *α*-(1→3)-mannooligosaccharides. Instead, we used a heterogeneous ligand-parallel reaction model (Smith *et al.*, 2003; Yaqub *et al.*, 2003; Mader *et al.*, 2004) and a two-state reaction model to study the binding kinetics. As shown in Table 2, the heterogeneous ligand-parallel reaction model indicated two binding sites. Similar affinity constants (*K*_d1_) were calculated for the *α*-(1→3)-mannooligosaccharides at the first binding site, although the order depended on size (mannopentaose<mannotetraose<mannotriose<mannobiose); however, the second parallel reaction affinity (*K*_d2_) clearly increased according to the order mannotetraose, mannobiose, mannopentaose and mannotriose. The differences among these affinities might have contributed to the variation in the sensorgrams. In contrast, the two-state reaction model gave similar affinity constants (*K*_d_) among the oligosaccharides, with values and an order similar to those of *K*_d1_.

### Docking simulation for mannotriose from *C. neoformans* and TPI from *S. aureus*

**Fig. 4. f4:**
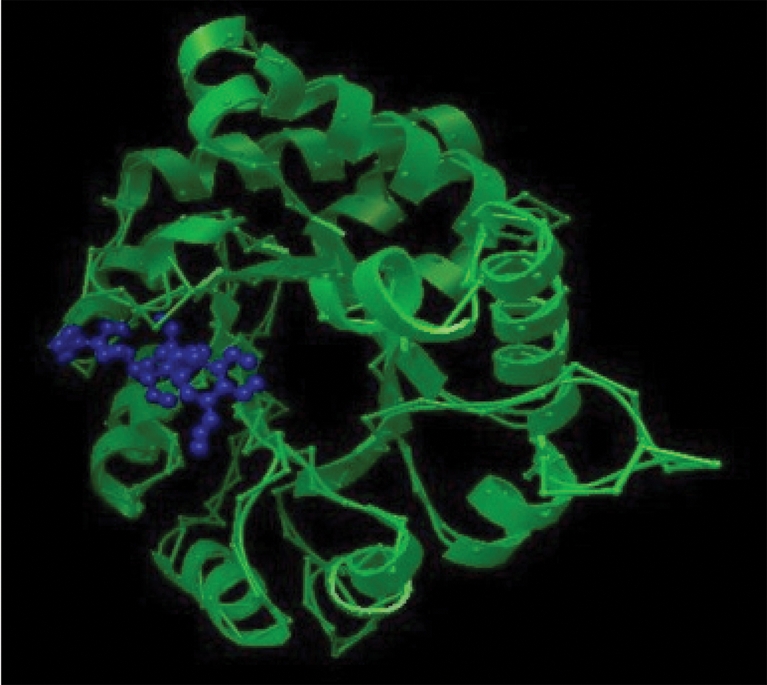
A docking model of TPI from *S. aureus* (green) and mannotriose (blue) constructed using AutoDock 3.05. The tertiary model structure of TPI was generated by swiss-model. TPI of *Bacillus stearothermophilus* (PDB code 2btmB) was used as the template.

We performed docking simulation analyses for *S. aureus* TPI (253 amino acids) with AutoDock 3.05 using a PDB file for TPI from *S. aureus* obtained by homology modelling. Several possible conformations were obtained, and the interaction between TPI and mannotriose near the substrate binding site was suggested (Fig. 4).

### Inhibition of TPI activity by *α*-(1→3)-mannooligosaccharides

**Fig. 5. f5:**
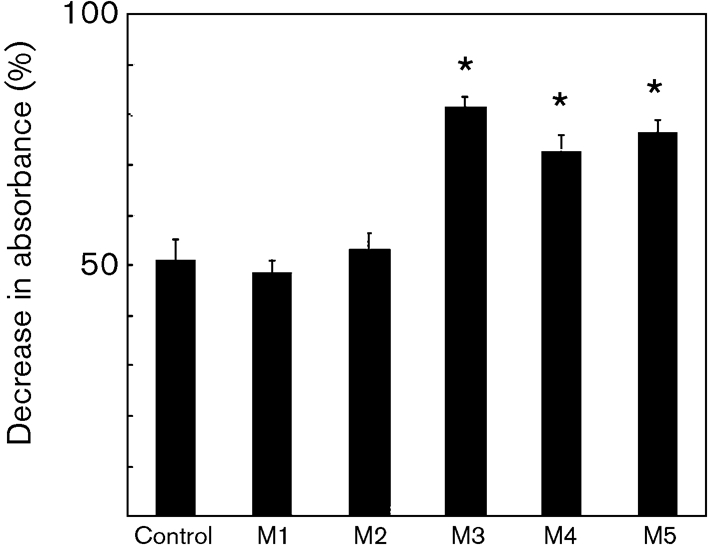
TPI inhibition assay using *α*-(1→3)-mannooligosaccharides. Mannooligosaccharides were added at a concentration of 100 μM. M1, mannose; M2, mannobiose; M3, mannotriose; M4, mannotetraose; M5, mannopentaose. The control did not contain these oligosaccharides. *, *P*<0.01.

**Fig. 6. f6:**
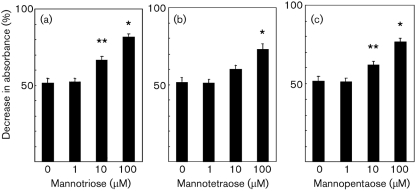
TPI assay in the presence of the indicated concentrations of mannooligosaccharides. (a) Mannotriose, (b) mannotetraose, (c) mannopentaose. *, *P*<0.01; **, *P*<0.05.

The *α*-(1→3)-mannooligosaccharides larger than mannotriose demonstrated inhibitory effects on TPI activity (Fig. 5). Furthermore, dose-dependent inhibition was observed with mannotriose, mannotetraose and mannopentaose (Fig. 6).

## DISCUSSION

Glycolytic enzymes have been found on the cell surfaces of certain bacteria and fungi. In addition to their roles in glycolysis, some glycolytic enzymes, such as d-glyceraldehyde-3-phosphate dehydrogenase, that occur both on the cell surface and in a secreted form have been shown to possess multi-functional properties, including adhesion and nutrient uptake (Modun & Williams, 1999; Bergmann *et al.*, 2004; Gatlin *et al.*, 2006; Terao *et al.*, 2006; Antikainen *et al.*, 2007; Egea *et al.*, 2007). Furthermore, interactions have been reported between glycolytic enzymes and human bioactive proteins, such as fibrinogen, plasminogen and fibronectin, and glycolytic enzymes expressed on microbial cell surfaces have become targets for the regulation of bacteria and fungi (Pancholi & Fischetti, 1992; Gil-Navarro *et al.*, 1997; Pancholi & Fischetti, 1998; Bergmann *et al.*, 2001).

Previously, we found that the bacterium *S. aureus* adheres to and kills the fungal pathogen *C. neoformans* (Saito & Ikeda, 2005). We identified TPI as a candidate adherence molecule on *S. aureus* that recognizes the mannan backbone in GXM, a major component of the capsule of *C. neoformans* (Ikeda *et al.*, 2007). The glycolytic enzyme TPI, which catalyses the reversible reaction between d-glyceraldehyde 3-phosphate and dihydroxyacetone phosphate, is located on the cell surface, although it is not as abundant as the cell surface glyceraldehyde-3-phosphate dehydrogenases. TPI is also thought to play a role in adhesion with the fungal pathogen *Paracoccidioides brasiliensis* (Pereira *et al.*, 2007).

In this study, we purified TPI from *S. aureus* and characterized its interaction with a series of *α*-(1→3)-mannooligosaccharides, with various degrees of polymerization, obtained from *C. neoformans*. TPI was purified from *S. aureus* surface proteins by salting out and hydrophobic, anion-exchange and gel-filtration column chromatography. The purified TPI yielded a single band in SDS-PAGE. SPR analysis of the interaction between TPI from *S. aureus* and GXM from *C. neoformans* showed that purified TPI interacted with GXM in a dose-dependent manner, providing evidence that staphylococcal TPI and cryptococcal GXM participate in the adherence between these two organisms. Subsequently, we examined the interactions between purified TPI and *α*-(1→3)-mannooligosaccharides derived from the backbone of GXM. Mannose did not display specific binding. During the first 60 s after injection, the SPR responses of mannobiose and mannotetraose were lower than those of mannotriose and mannopentaose, respectively. However, between 60 and 600 s, the responses of mannobiose and mannotetraose were greater than those of mannotriose and mannopentaose, respectively. For the kinetic analyses, two fitting models were selected: the heterogeneous ligand-parallel reaction model and the two-state reaction model. Two binding affinities, *K*_d1_ and *K*_d2_, were calculated from the former model; the *K*_d1_ values were similar among the oligosaccharides, whereas the second reaction appeared to occur with significantly different affinities for the oligosaccharides. This suggests that TPI has at least two binding sites with different affinities. In the two-state reaction model, binding is followed by a conformational change (A+B↔AB↔AB_x_). The affinity increased slightly with increasing number of mannose residues. The sensorgrams showed an increasing response according to the order mannotetraose, mannobiose, mannopentaose and mannotriose. We considered the heterogeneous ligand-parallel reaction model to be a better fit for the interaction of *α*-(1→3)-mannooligosaccharides, especially even-numbered oligosaccharides, and TPI from *S. aureus*.

The values for *K*_d2_ were higher for odd-numbered compared to even-numbered oligosaccharides. Based on the *K*_d2_ and the shape of the sensorgrams, the participation of the second affinity may be less significant for the odd-numbered oligosaccharides. Previously, we proposed three-dimensional models of the oligosaccharides (Ikeda *et al.*, 2007). The characteristic structure with a curve appeared in oligosaccharides larger than mannotriose. From the curved chemical structures, we inferred that common binding sites within oligosaccharides of a size equal to or greater than that of triose could be postulated. In the docking simulation, a conformation was suggested that included the formation of hydrogen bonds to Lys^11^, Asn^13^, Gly^175^, Ser^215^ and Lys^217^. Furthermore, other binding pockets, which would not affect enzyme activity, could be predicted for biose and tetraose. In particular, mannobiose may bind TPI in a multiple-binding manner; therefore, the affinity of mannobiose was higher than that of mannopentaose or mannotriose. Comparing mannobiose and mannotetraose, mannotetraose displayed a higher affinity (*K*_d2_ 1.46×10^−8^) than mannobiose (*K*_d2_ 5.37×10^−6^). Overall, mannotetraose showed the highest affinity among the oligosaccharides tested.

The results of computational docking and inhibition assays for TPI activity revealed the possibility that mannooligosaccharides larger than mannotriose may bind to *S. aureus* TPI near the substrate-binding site. The structural model of TPI suggests that this enzyme may have multiple binding pockets and that they bind to mannose residues.

It is important to characterize the binding of TPI proteins to *C. neoformans* cells, including their localization. For this purpose, it is necessary to prepare anti-TPI antibodies. The preparation of sufficient TPI, including recombinant TPI, should be helpful.

Since mannose plays an important role in recognizing molecules involved in infection and innate immunity (van de Wetering *et al.*, 2004; Dommett *et al.*, 2006), the presence of unknown substances that interact with TPI is expected. Therefore, TPI may be a multifunctional protein that binds with eukaryotic glycoproteins. The use of carbohydrates as anti-adhesion drugs for the regulation of pathogens has also been reported (Liu *et al.*, 2000; Sharon, 2006). The elucidation of the role of TPI as a virulence factor of *S. aureus* may prove valuable for screening novel anti-infective agents.

## Figures and Tables

**Table 1. t1:** Purification of TPI from *S. aureus*

**Purification step**	**Total protein (mg)**	Total TPI activity (units*)	**Specific activity (units mg^−1^)**	**Yield (%)**	**Purification (fold)**
Crude extract	10.5	57.3	5.5		1
Salting out	5.3	35.2	6.6	61	1.2
Hydrophobic interaction	5.4×10^−2^	66.7	1.2×10^3^	116	220
Anion exchange	5.7×10^−3^	68.1	1.2×10^4^	119	2200
Gel filtration	1.7×10^−3^	22.2	1.3×10^4^	39	2400

*A unit is defined as the amount of enzyme required to cause a change in *A*_340_ of 0.002 in 1 min in the spectrophotometric assay utilizing glyceraldehyde 3-phosphate as substrate.

**Table 2. t2:** Affinities of *α*-(1→3)-mannooligosaccharides to TPI from *S. aureus*

Oligosaccharide*	**Heterogeneous ligand-parallel reaction**		**Two-state reaction *K*_d_ (M)**
	***K*_d1_ (M)**	***K*_d2_ (M)**	
M2	4.77×10^−3^	5.37×10^−6^	4.45×10^−3^
M3	2.02×10^−3^	1.24×10^−4^	1.44×10^−3^
M4	3.14×10^−4^	1.46×10^−8^	5.17×10^−4^
M5	2.08×10^−4^	1.86×10^−5^	2.31×10^−4^

*M2, mannobiose; M3, mannotriose; M4, mannotetraose; M5, mannopentaose.

## Abbreviations

- GXM, glucuronoxylomannan
- SPR, surface plasmon resonance
- TPI, triosephosphate isomerase
